# Inverse forgetting in unconscious episodic memory

**DOI:** 10.1038/s41598-022-25100-w

**Published:** 2022-11-29

**Authors:** Luca Pacozzi, Leona Knüsel, Simon Ruch, Katharina Henke

**Affiliations:** 1 Institute of Psychology, University of Bern, 3012 Bern, Switzerland; 2 Institute for Neuromodulation and Neurotechnology, Department of Neurosurgery and Neurotechnology, University Hospital and University of Tuebingen, 72076 Tübingen, Germany

**Keywords:** Neuroscience, Cognitive neuroscience, Learning and memory

## Abstract

Forming memories of experienced episodes calls upon the episodic memory system. Episodic encoding may proceed with and without awareness of episodes. While up to 60% of consciously encoded episodes are forgotten after 10 h, the fate of unconsciously encoded episodes is unknown. Here we track over 10 h, which are filled with sleep or daytime activities, the retention of unconsciously and consciously experienced episodes. The episodes were displayed in cartoon clips that were presented weakly and strongly masked for conscious and unconscious encoding, respectively. Clip retention was tested for distinct clips directly after encoding, 3 min and 10 h after encoding using a forced-choice test that demands deliberate responses in both consciousness conditions. When encoding was conscious, retrieval accuracy decreased by 25% from 3 min to 10 h, irrespective of sleep or wakefulness. When encoding was unconscious, retrieval accuracy increased from 3 min to 10 h and depended on sleep. Hence, opposite to the classic forgetting curve, unconsciously acquired episodic memories strengthen over time and hinge on sleep on the day of learning to gain influence over human behavior.

## Introduction

The most replicated finding in research on episodic memory, i.e., the memory of personally experienced episodes, is a fall of retrieval accuracy with passing time. This decay is seen when retention is measured over a period of minutes and hours. The classic Ebbinghaus forgetting curve is a negatively accelerating function consisting of an initial rapid decline in retrieval accuracy followed by a slow decay^1^. The shape of the forgetting curve varies as a function of the kind of the given learning material and the applied retrieval task. Yet, the curve points always into one direction: downwards^2^. This is also true for traditional implicit or non-declarative memory tasks (word stem completion and word fragment completion tasks), where memory retrieval is assessed without reference to the (conscious) encoding situation^3,4^. On implicit memory tasks, participants engage in a seemingly memory-unrelated task with their performance reflecting prior learning.

Using implicit memory tasks given between 3 and 25 min following subliminal (unconscious) paired-associative learning, our group has demonstrated that item combinations are processed through the episodic memory system and are flexibly represented^5–12^, evincing an unconscious form of episodic memory^13^. Yet, the long-term consolidation trajectory of subliminally (unconsciously) acquired episodic memories has not been studied. Consolidation is a process that refers to the implementation of new memories in the brain, both at the cellular/synaptic level and at the systems level. Mechanisms that strengthen synapses, such as long-term potentiation, are thought to drive memory consolidation^14^. The aim of the present research was to compare the consolidation trajectory of unconsciously versus consciously acquired episodic memories over 10 h with identical learning material and retrieval formats. We hypothesized that both consciously and unconsciously acquired episodic memories would be retained over 10 h because both have been shown to be processed through the episodic long-term memory system^13^. Long-term effects of unconsciously acquired episodic memories on decision making have been reported previously^10,12,15^. The episodic memory system makes use of a fast-acting associative encoding and long-term storage machinery centered on the hippocampus, a brain structure in the medial temporal lobe^13,16,17^. Therefore, we expected both consciously and unconsciously acquired memories to outlast several hours.

The hypothesized long-term retention of subliminally presented material seems counterintuitive given the literature on subliminal processing which indicates that subliminal stimuli affect human behavior over 1 s at most^18–23^. However, these short-lived subliminal retention effects were obtained with single item priming, using familiar stimuli such as words. Because the subliminal priming of familiar items recruits the neocortex alone—and not the hippocampus^5^—and because the neocortex is a slow learner^17^, the resulting neocortical memory traces decay rapidly. However, when presenting novel multi-item displays subliminally^5–9,12^, the encoding process recruits the episodic memory system including the hippocampus. This subliminal presentation method yielded retrieval effects over 25 min^10^. Here, we probe the retention over 10 h, using subliminal cartoon clips, whose encoding and 3.5 min-retrieval had engaged the hippocampus in relation to retrieval success in an earlier study^12^. Because these cartoon clips are novel and complex, they call upon the hippocampus, which might provide—so our hypothesis—the necessary plasticity mechanisms to store the clips over hours.

Interestingly, when novel and complex stimuli are being presented subliminally (unconsciously)—but not supraliminally (consciously)—their processing requires several minutes before the formed memories can influence reaction times or response accuracy in retrieval tasks [e.g.,^10^^,^^11^^,^^12^]. A likely reason for this extra time requirement of subliminally versus supraliminally formed memories might be the weak sensory input emitted by the subliminal presentation, which elicits weak and sparse activation patterns in the brain^12^. We speculate that these activation patterns dissolve unless they undergo an immediate consolidation process^10–12^. This consolidation process likely includes hippocampal long-term potentiation^14^ and neural replay, whereby the sequence of neuronal firing during encoding is repeated to strengthen connections between neurons coding for different aspects of an episode^24–28^. In fact, more neural replay has been observed in humans for supraliminally (consciously) acquired memories that were weakly versus strongly encoded^28^. The same has been found in rodents^29^.

Night sleep may benefit newly formed memories more than wake consolidation because much evidence indicates that hippocampal memory traces undergo a consolidation process during deep sleep^30–32^. When learning is followed by a sleep-filled versus sleep-free retention interval, the learning material is better retained over 24 h^33–35^. Interestingly, supraliminally (consciously) acquired weak versus strong memories undergo a privileged sleep-assisted consolidation process^36–41^. Hence, it appears that weak memory traces that are in particular need of strengthening have privileged access to the consolidation machinery. We therefore hypothesize that the longevity of subliminally (unconsciously) formed memories, due to their weakness, will depend on sleep to a larger extent than supraliminally (consciously) formed episodic memories.

With the current experimental design, we compared between-subjects the consolidation trajectories of subliminally (unconsciously) and supraliminally (consciously) encoded episodes. We presented strongly and weakly masked cartoon clips for unconscious and conscious encoding, respectively. Each clip presented a scene with a visually impenetrable hiding place and five animals that entered and left the hiding place consecutively. Animals moved through the hiding place and left it immediately or lingered inside, potentially meeting other animals. Participants’ task was to encode the times of the animals’ entrances and exits to infer which animals resided in the hiding place simultaneously. Drawing inferences while encoding animal trajectories requires Piagetian object permanence^42^ and working memory.

Because the successful encoding of animal encounters in the hiding place relies upon the constant mental updating of each animal’s whereabouts to draw inferences, the encoding process poses high demands on the hippocampus-dependent episodic buffer of working memory^43,44^. That this encoding task activates the visuospatial working memory has been confirmed by Schneider et al.^12^, who experimented with the same cartoon clips. Schneider et al.^12^ found the hippocampus and the right prefrontal cortex, both areas supporting visuospatial working memory [e.g.,^45^^,^^46^^,^^47^], to increase their activation during conscious and unconscious inference making during clip encoding. Importantly, the activity increase in these regions recorded during conscious/unconscious inference making correlated with the delayed retrieval performance^12^. This shows that the successful drawing of inferences is mandatory for a good retrieval performance: without inference making, the information requested on the retrieval test is not available. Furthermore, research with other inference tasks has emphasized the important role of working memory and the prefrontal cortex^48–53^. Inferential reasoning has been shown to depend on medial temporal and prefrontal brain areas even when information processing and the drawing of inferences is unconscious^8,12,54^. Therefore, we assume that a well-functioning visuospatial working memory is prerequisite to encoding animal encounters in a visually impenetrable hiding place.

**Figure 1:**
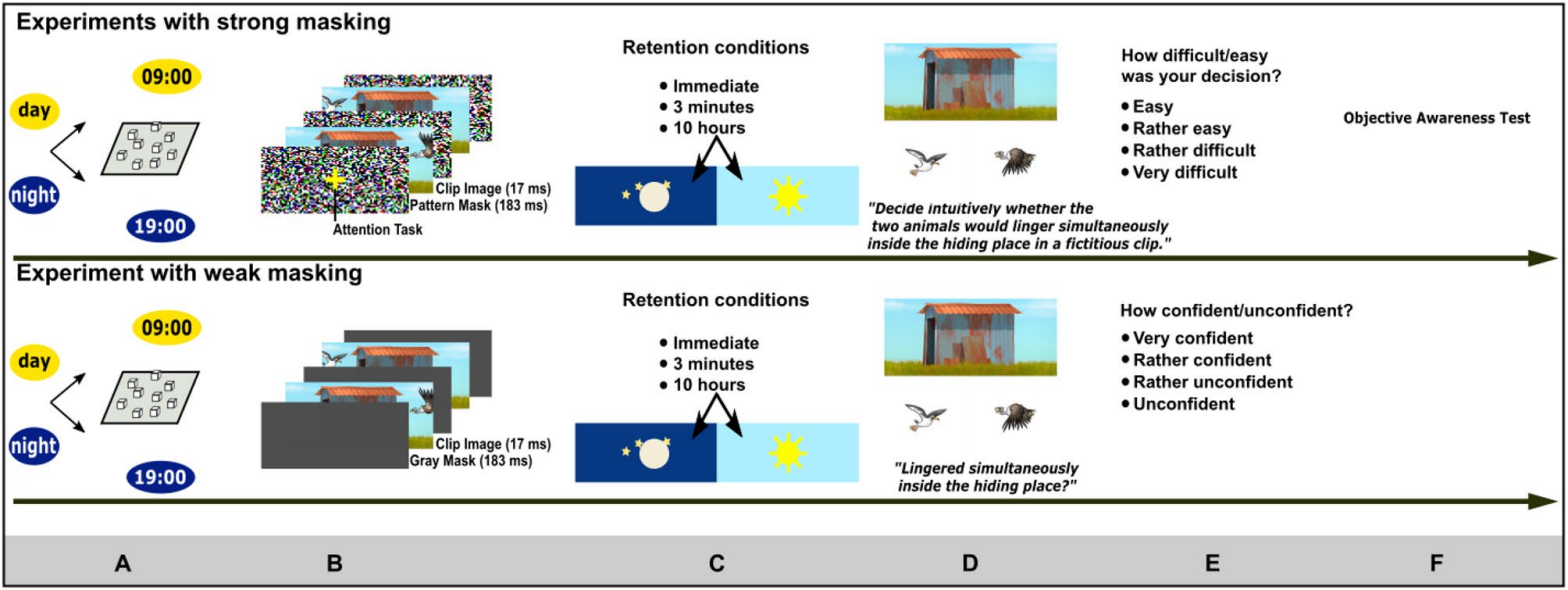
Experimental design. (**a**). Encoding took place in the morning or in the evening. Before encoding, participants performed the Corsi block-tapping test backwards to assess the visuospatial working memory capacity. (**b**) Clips were presented strongly masked for unconscious encoding and weakly masked for conscious encoding. When masking was strong, participants performed a central attention task during clip presentation. Every cartoon frame was repeated three times in sequence but for display purposes, a frame is depicted only once. (**c**–**e**) Six of the 18 displayed clips were randomly assigned to each of the three retention conditions (**c**). Retrieval trials (**d**) and confidence/difficulty ratings (**e**) were prompted immediately after clip offset or following three minutes or following 10 h. (**f)** When clips were presented subliminally, we administered an objective test of clip awareness at the end of the experiment.

**Figure 2:**
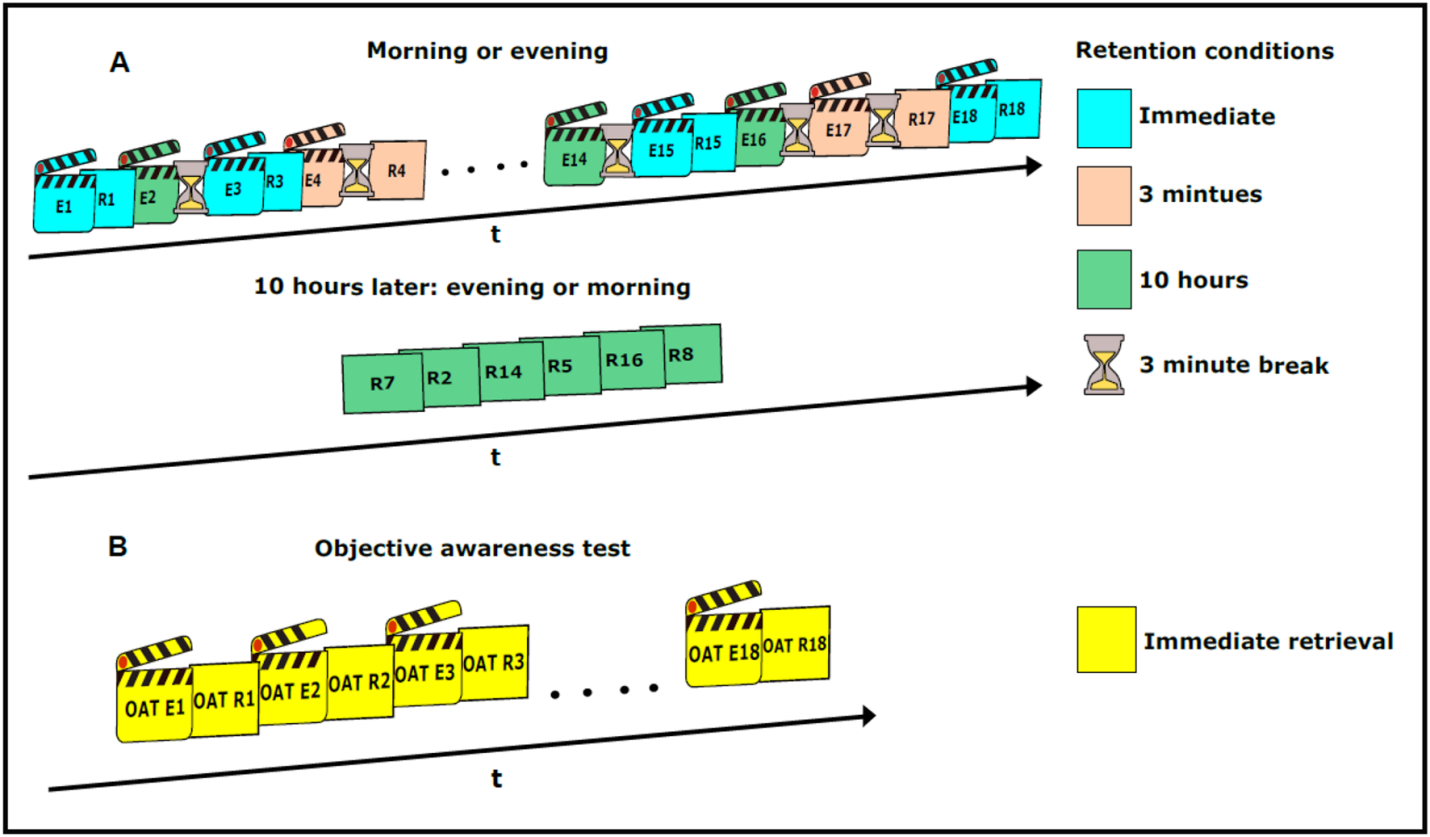
Experimental procedure. (**A**) Eighteen cartoon clips were presented subliminally/supraliminally for unconscious/conscious encoding. A 3-min silent break followed clip presentations both in the 3-min and 10-h retrieval condition. (**B**) We ran an objective test of clip awareness with 18 strongly masked clips at the end of the experiment. Here, retrieval testing was prompted immediately after clip offset. OAT, objective awareness test; E, encoding; R, retrieval; 1–18: number of cartoon clip.

**Figure 3:**
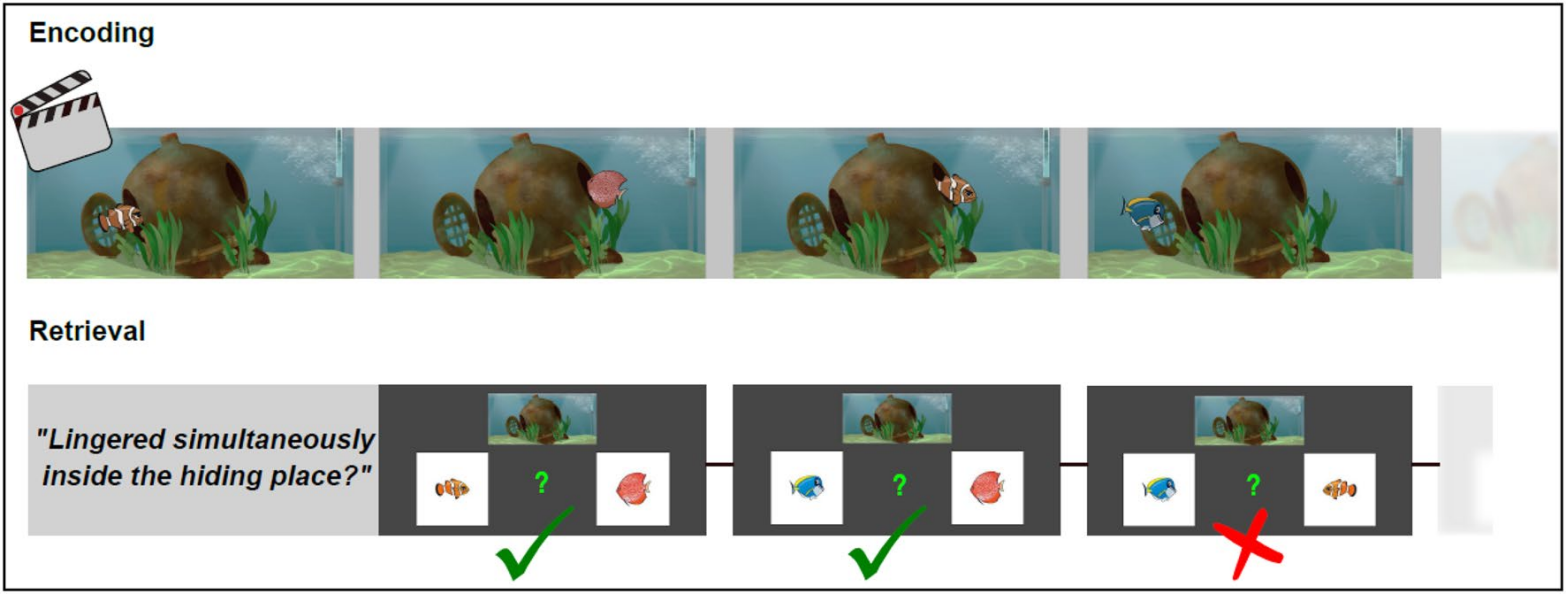
Retrieval task. Each cartoon clip featured five animals that crossed the scene consecutively. An animal could either move straight through the hiding place or linger inside for an arbitrary dwelling time and meet other animals that had entered before or would arrive later. Participants could not see the inside of the hiding place, i.e., they could not see the animals linger inside. Therefore, they needed to observe the animals’ entrances and exits to keep a mental record of which animals lingered simultaneously inside the hiding place. When clips were presented subliminally, participants watched the flickering display (induced by the masks) and performed a central attention task. Following supraliminal clip presentations for conscious encoding, participants’ retrieval instruction was to decide, based on conscious memory, whether the two animals had lingered simultaneously inside the hiding place or not. Following subliminal clip presentations for unconscious encoding, participants’ retrieval instruction was to decide intuitively whether the two animals would linger simultaneously inside the depicted hiding place in a fictitious clip.

In these experiments, the outputs of working memory needed to be retrieved immediately following encoding or retained over 3 min or 10 h (Figs. 1, 2, and 3). Of the 18 clips presented for encoding, 6 clips were assigned to each retention condition (immediate, 3 min, 10 h; varied within-subjects). The 10 h retention period was filled either with night sleep or daytime activities. A retrieval trial consisted of the unmasked presentation of a clip’s hiding place plus two animals that had featured in the clip. Following supraliminal clip presentations for conscious encoding, participants’ retrieval instruction was to decide, based on conscious memory, whether the two animals had lingered simultaneously inside the hiding place or not (Fig. 1D). The experiments with subliminal clip presentation kept participants naïve to the fact that they are being exposed to subliminal stimuli. It has previously been shown that uninformed participants are more susceptible to subliminal information^55,56^. Following subliminal clip presentations for unconscious encoding, participants’ retrieval instruction was to decide intuitively whether the two animals would linger simultaneously inside the depicted hiding place in a fictitious clip. After every response, participants indicated their confidence in their response (supraliminal version) or their felt ease of giving the response (subliminal version) using a 4-point scale (Fig. 1E). Following the subliminal version of the experiment, participants took an objective test of clip awareness (Fig. 1F) to assess the effectiveness of the masking protocol (Fig. 2B). Because the design of the encoding task requires a strong visuospatial working memory, we assessed the conscious visuospatial working memory capacity in all participants using the Corsi Block-Tapping Test (Fig. 1A).

## Results

### Experiment with strong masking for unconscious encoding

The experiment with strong masking (pattern masks) for unconscious clip encoding was run in 73 participants. Five participants were excluded from the data analysis due to compliance failure with given instructions (see “Methods and material”). Participants were allocated post-hoc to a high- or a low-working-memory performance group (*High-Low¸* by median-split; N = 27 above median; N = 41 less than or equal to the median) (Supplementary Table 1). We decided to split the sample based on the median because of the unequal distribution of the working memory scores. The unbalanced cell counts (see Supplementary Table 3) did not allow for the inclusion of the working memory data as a linear predictor in the computed models. Of note, the median and the mode of the working memory scores were equal, which means that only participants scoring above the most common score were assigned to the group of high-working-memory performers.

We computed an analysis of variance (ANOVA) that included the within-subjects factor Retention (immediate; 3 min; 10 h) and the between-subjects factors Day-Night (day group: wake consolidation during daytime activities; night group: sleep consolidation during nighttime) and High-Low (high- vs. low-working-memory performance group). The dependent variable was retrieval accuracy (percentage correct). The ANOVA was followed by t-tests to ascertain whether retrieval accuracy exceeds chance-level (50% correct). To quantify the evidence for an above-chance retrieval performance, we computed Bayes Factors using the R function^57^ provided by Baguley^58^ with a half-normal prior distribution and a mode of 0% (chance level) and a standard deviation of 5% (based on^10^).

#### High-working-memory performers retained clips over 10 h

The repeated-measures ANOVA yielded a significant main effect of High-Low (*F* (1, 64) = 8.529, *p* = 0.005, ${\eta }_{\rho }^{2}$ = 0.118), indicating that clip retention was better in high- versus low-working-memory performers. The intercept for high-working-memory performers exceeded chance-level (*F* (1, 25) = 13.033, *p* = 0.001, ${\eta }_{\rho }^{2}$ = 0.343). However, the ANOVA yielded neither a significant intercept over all participants (*F* (1, 64) = 1.087, *p* = 0.301, ${\eta }_{\rho }^{2}$ = 0.017) nor significant main effects of Retention (*F* (2, 128) = 2.680, *p* = 0.072, ${\eta }_{\rho }^{2}$ = 0.04) and Day-Night (*F* (1, 64) = 2.362, *p* = 0.129, ${\eta }_{\rho }^{2}$ = 0.036), nor significant interactions (all *p* > 0.295) (Supplementary Table 1).

Based on previous research emphasizing the role of working memory for the drawing of inferences^12,48–53^ and because we had expected that minutes to hours of consolidation would be necessary to strengthen the memory traces to influence conscious decision-making^10,12,15^, we performed one-tailed post-hoc t-tests between high- versus low-working memory performers in the 10 h retrieval condition. These analyses showed a significant difference in retrieval accuracy between high- versus low-working memory performers in the 10 h retrieval condition (Bonferroni-adjusted, *α* = *0.016,* t (66) = − 2.431, *p* = 0.009, one-tailed), but in no other condition (both *p* > 0.028). Because we had also expected retrieval accuracy in the high-working-memory performers to exceed the chance-level (50%) at minutes and at hours following encoding, but not immediately following encoding^10–12^, we computed one-tailed one-sample t-tests to compare retrieval accuracy against chance-level for the 3 min and the 10 h retrieval interval. High-working-memory performers exhibited accuracy scores of 51.71% (95% CI [50.04, 53.39]) and 53.71% (95% CI [51.24, 56.19]) for the 3 min and 10 h interval, respectively. Clip retention was significantly above chance-level at 3 min (Bonferroni-adjusted, *α* = *0.025, t* (26) = 2.100, *p* = 0.023, one-tailed) and at 10 h (Bonferroni-adjusted, *α* = *0.025, t* (26) = 3.083, *p* = 0.0025, one-tailed). Bayes Factors for the 3 min and 10 h condition were BF_10_ = 2.68 and BF_10_ = 41.55, respectively, revealing anecdotal evidence for H_1_ regarding the 3 min condition and strong evidence for H_1_ regarding the 10 h condition.

#### Night sleep promotes clip retention in high-working-memory performers

**Figure 4:**
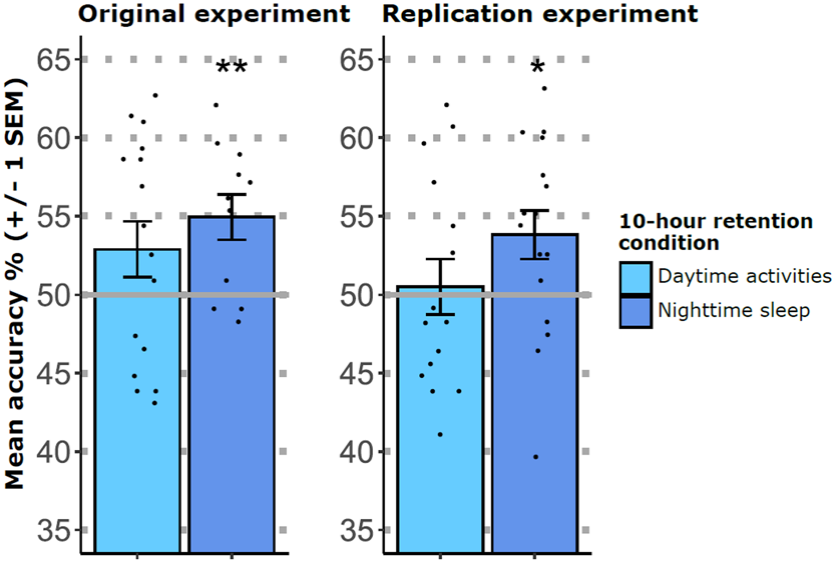
Retrieval accuracy in the 10-h retention condition following strong masking. In the original experiment with strong masking, the high-working-memory performers yielded an above-chance retrieval accuracy, if the 10-h retention interval was filled with night sleep. Also in the replication experiment with strong masking, the high-working-memory performers yielded an above-chance retrieval accuracy, if the 10-h retention interval was filled with night sleep. Asterisks represent significant one-sample t-tests against chance-level (*α* = *.025;* *p < 0.025, BF_10_ = 9.2; **p < .01, BF_10_ = 114.28; one-tailed). Error bars represent the standard error of the mean (SEM).

Based on previous findings regarding the consolidation of weakly versus strongly coded memories, we had hypothesized that a sleep- versus daytime-consolidation would be better for the retention of unconsciously encoded clips^36–41^. To detect an interaction between Retention and Day-Night, we computed a repeated-measures ANOVA with the retrieval data of the high-working-memory performers. This analysis yielded neither a significant main effect for Day-Night (*F* (1, 25) = 0.982, *p* = 0.331, ${\eta }_{\rho }^{2}$ = 0.038) nor a significant interaction between Retention and Day-Night (*F* (2, 50) = 0.428, *p* = 0.654, ${\eta }_{\rho }^{2}$ = 0.017). To follow up on our directed hypothesis, we now computed one-tailed one-sample t-tests against chance-level (50%) for the day and for the night group. Indeed, high-working-memory performers exhibited above-chance accuracy scores in the 10 h condition filled with night sleep (54.93%, 95% CI [51.70, 58.17], Bonferroni-adjusted, *α* = *0.025, t* (10) = 3.401, *p* = 0.0035, one-tailed) but not in the 10 h condition filled with daytime activities (52.87%, 95% CI [49.08, 56.66], Bonferroni-adjusted, *α* = *0.025, t* (15) = 1.616, *p* = 0.0635, one-tailed). Bayes Factors for night sleep and daytime activities were BF_10_ = 114.28 and BF_01_ = 1.99, respectively, which is substantial evidence that night sleep promoted memory consolidation (Fig. 4). It should be noted that the absence of a significant main effect Day-Night in the above reported ANOVA suggests that the day and night samples did not differ systematically in retrieval performance (subject bias) but only with respect to the 10 h retention condition.

#### Difficulty ratings were unrelated to retrieval accuracy

After every retrieval trial, participants were asked how difficult it was to make the decision of whether the two animals might linger simultaneously in a fictitious clip (1: easy; 2: rather easy; 3: rather difficult; 4: very difficult). We related this predictor variable to the dichotomous outcome variable accuracy (correct versus incorrect responses). This binary logistic regression was run for the 10 h condition including night sleep in high-working-memory performers. The difficulty rating was not predictive of retrieval accuracy: The unstandardized Beta weight for the constant was *β* = -0.375, SE = 0.271, Wald = 1.912, *p* = 0.167. The unstandardized Beta weight for the predictor was *β* = 0.066, SE = 0.089, Wald = 0.553, *p* = 0.457. The estimated odds ratio was [Exp (*β*) = 1.068, 95% CI (0.897, 1.272)]. Hence, the metamemory-based, subjectively experienced ease of making the decision at test was unrelated to response accuracy, which suggests that the retrieval was completely unconscious.

#### Measures of sleep-related behavior

When a night intervened encoding and retrieval in the 10 h condition, participants wore an actigraphy device^59^ that measures environmental brightness and the participants’ movement-related behavior to determine the total time in bed, the assumed actual sleep duration and a sleep fragmentation index. Four participants were excluded from the analyses due to recording failure. The mean total time in bed was 7.5 h (std: ± 0.52) and the mean duration of sleep was 6.3 h (std: ± 0.67), which indicates compliance with the given sleep-related instructions. Sleep duration and sleep fragmentation were unrelated to retrieval accuracies (all *p* > 0.282).

### Replication experiment with strong masking for unconscious encoding

We replicated the above reported experiment with strong masking to ascertain that high-working-memory performers can encode clips and retrieve them following 10 h filled with sleep. We included only participants (N = 31) with a high visuospatial working memory capacity, meaning only subjects with a working memory score higher than the median obtained in the first experiment with strong masking. Hence, we computed t-tests of retrieval accuracy against chance-level for the 10 h retention condition filled with sleep or filled with daytime activities. We also computed the Bayes Factors to quantify the strength of evidence for memory performance above chance-level. The Bayes Factors were calculated using the R function^57^ provided by Baguley^58^, with the same parameters as in the previous experiment. Five participants were excluded from the analyses due to compliance failure with given instructions (see “Methods and material”).

Retrieval accuracy in the 10 h condition filled with sleep exceeded chance-level (memory accuracy: 53.81%, 95% CI [50.50, 57.11], one-sample t-test, Bonferroni-adjusted, *α* = *0.025, t* (15) = 2.454, *p* = 0.0135, one-tailed). However, retrieval accuracy in the 10 h condition filled with daytime activities did not differ from chance level (memory accuracy: 50.53%, 95% CI [46.76, 54.29], Bonferroni-adjusted, *α* = *0.025, t* (13) = 0.3, *p* = 0.384, one-tailed) (Fig. 4). The Bayes Factors for the sleep and daytime activities conditions were BF_10_ = 9.2 and BF_01_ = 0.42, respectively, which is evidence for an above-chance accuracy in the sleep condition.

#### Difficulty ratings were unrelated to retrieval accuracy

We related the difficulty ratings as predictor variable to the dichotomous outcome variable accuracy (correct versus incorrect responses). This binary logistic regression showed that the difficulty ratings were not predictive of retrieval accuracy in the 10 h condition filled with sleep. The unstandardized Beta weight for the constant was *β* =  − 0.537, SE = 0.229, Wald = 5.483, *p* = 0.019. The unstandardized Beta weight for the predictor was *β* = 0.131, SE = 0.073, Wald = 3.260, *p* = 0.071. The estimated odds ratio was [Exp (*β*) = 1.140, 95% CI (0.989, 1.314)]. Hence, the metamemory-based, subjectively experienced ease of making the decision at test was unrelated to response accuracy, which suggests that the retrieval was completely unconscious.

#### Measures of sleep-related behavior

Two participants were excluded from the analyses due to recording failure. Mean total time in bed was 8.38 h (std: ± 0.36), indicating compliance with given instructions. The mean duration of assumed actual sleep was 7.68 h (std: ± 0.46). Sleep duration and sleep fragmentation were unrelated to retrieval accuracy (all *p* > 0.279).

### Objective and subjective awareness measures suggest unconscious clip encoding

At this point, participants were fully informed of subliminal clips. The awareness test was run after the main experiment with another 18 cartoon clips that were presented strongly masked, applying the same experimental procedure as in the main experiment. Yet, the retrieval trials were given without retention interval and retrieval instructions were direct rather than indirect. Immediately following the presentation of a clip, participants scored the visibility of the clip using a 4-point perceptual awareness scale ^60^ with the levels: (1) no clip awareness at all; (2) a feeling that something was present, either static or moving, (3) an impression of the scene or animals, (4) a clear image of the scene and animals. The 10 retrieval trials followed this subjective awareness rating. The retrieval instruction required participants to indicate—based on what they had just seen—whether the two presented animals had lingered simultaneously inside the hiding place or not. The evaluation of the subjective visibility ratings and the retrieval responses indicated that participants had processed the strongly masked clips without any conscious awareness (see supplement for details).

### Experiment with weak masking for conscious encoding

The experiment with weak masking (grey masks) for conscious clip encoding was run in 36 participants. To examine the influence of visuospatial working memory capacity on clip encoding/retrieval, participants took the Corsi Block-Tapping test and were allocated post-hoc to a high- or a low-working-memory performance group (*High-Low¸* by median-split; N = 16 above median; N = 20 equal to or below median) (Supplementary Table 1). Because of the uneven distribution of the working memory scores, we could not include this variable in the model as a linear predictor (Supplementary Table 3). We computed an ANOVA that included the within-subjects factor Retention (immediate; 3 min; 10 h) and the between-subjects factors Day-Night and High-Low (high- versus low-working-memory performance group). The dependent variable was retrieval accuracy (percentage correct). The ANOVA was followed by t-tests to ascertain whether retrieval accuracy exceeds chance-level (50% correct).

#### Retrieval performance drops significantly at ten hours following encoding

For retention conditions immediate, 3 min and 10 h, memory accuracy was 87.87% (95% CI [84.27, 91.48]), 87.56% (95% CI [84.47, 90.66]) and 66.04% (CI [61.21, 70.87]), respectively. Hence, the mean retrieval performance had dropped by 25% from the 3 min to the 10 h retention condition.

The ANOVA revealed that there was no main effect of Day-Night (*F* (1, 32) = 0.377, *p* = 0.544, ${\eta }_{\rho }^{2}$ = 0.012), no main effect of High-Low (*F* (1, 32) = 0.002, *p* = 0.967, ${\eta }_{\rho }^{2}$ = 0.00) and none of the interactions reached significance (all *p* > 0.146). Hence, night sleep versus daytime activities and visuospatial working memory performance exerted no differential effects on retrieval accuracy. However, there was a significant main effect of Retention (*F* (2, 64) = 83.948, *p* < 0.001, ${\eta }_{\rho }^{2}$ = 0.724). Post-hoc t-tests showed that retrieval accuracy was significantly better immediately after encoding (Bonferroni-adjusted, *α* = *0.016*, *t* (35) = 9.368, *p* < 0.001) and 3 min after encoding (Bonferroni-adjusted, *α* = *0.016, t* (35) = 10.620, *p* < 0.001) versus 10 h after encoding (descriptive results Supplementary Table 1). Yet, retrieval accuracy still exceeded the chance level of 50% in the 10 h condition (*t* (35) = 6.740, *p* < 0.001, 2-tailed).

#### Confidence ratings were related to retrieval accuracy

After every retrieval trial, participants were asked how confident they were with respect to whether the two animals lingered simultaneously in the hiding place (1: very confident; 2: rather confident; 3: rather unconfident; 4: unconfident). We related the confidence ratings as a predictor variable to the dichotomous outcome variable accuracy (correct versus incorrect responses). Because retrieval accuracy was above chance in each retention condition, we pooled trials. The binary logistic regression indicates that the predictor variable contributes significantly to the model. The unstandardized Beta weight for the constant was *β* = -3.653, SE = 0.090, Wald = 1635.743, *p* =  < 0.001. The unstandardized Beta weight for the predictor was *β* = 1.074, SE = 0.036, Wald = 901.451, *p* =  < 0.001. The estimated odds ratio was [Exp (*β*) = 2.928, 95% CI (2.730, 3.141)]. Hence, these metamemory-based confidence judgements predicted retrieval accuracy as would be expected for conscious episodic memory.

#### Measures of sleep-related behavior

Mean total time in bed was 7.6 h (std: ± 0.42) and the mean duration of assumed actual sleep was 6.5 h (std: ± 0.46), which indicates compliance with the given instructions. Sleep duration and sleep fragmentation were unrelated to retrieval accuracy (all *p* > 0.105).

### Comparing the consolidation trajectories between consciousness conditions

In the previous sections, we have reported that retrieval accuracy improved over 10 h when encoding was unconscious, but decreased when encoding was conscious. Here, we compared the consolidation trajectories of unconsciously and consciously acquired memories directly. To this aim, we collapsed and z-transformed scores for the retrieval accuracy over the three retention conditions of each experiment. For the original experiment with strong masking and for the experiment with weak masking, we included only the high-working-memory performers (N = 27, N = 16, respectively). For the replication experiment with strong masking (where only high-working-memory performers were included; N = 31), we included the entire sample.

#### Opposite consolidation trajectories after conscious versus unconscious encoding

**Figure 5:**
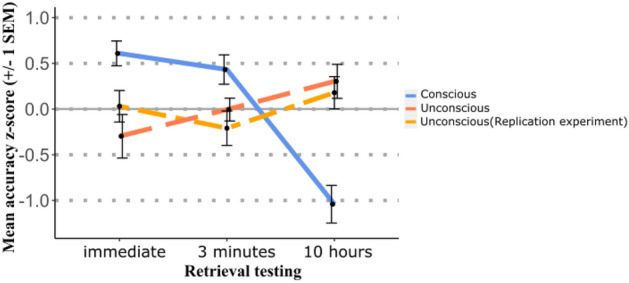
Opposite consolidation trajectories for conscious versus unconscious encoding. Z-transformed scores for the retrieval accuracy are plotted for each retention condition for the two experiments with strong masking (original and replication experiment) and the experiment with weak masking. While retrieval accuracy increased over 10 h in the two experiments with strong masking, retrieval accuracy dropped in the experiment with weak masking, evincing opposite consolidation trajectories for the two consciousness conditions. Error bars represent the standard error of the mean (SEM).

We computed a repeated-measures ANOVA with the within-subjects factor Retention and the between-subject factors Day-Night and Consciousness (original experiment with strong masking, replication experiment with strong masking, experiment with weak masking), with the z-transformed scores for the retrieval accuracy as the dependent measure. The interaction of Retention with Consciousness was significant (*F* (4, 136) = 8.494, *p* < 0.001, ${\eta }_{\rho }^{2}$ = 0.200). While the retrieval accuracy increased over 10 h in the two experiments with strong masking, retrieval accuracy dropped in the experiment with weak masking, evincing opposite consolidation trajectories for the two consciousness conditions (Fig. 5).

## Discussion

Much evidence suggests that episodic encoding may proceed with and without awareness of episodes. Yet, the long-term consolidation trajectory of unconsciously acquired episodic memories remains unstudied. We therefore tracked over 10 h, which were filled with night sleep or daytime activities, the retention of unconsciously and consciously encoded episodes displayed in cartoon clips. When encoding was conscious, retrieval accuracy decreased by 25% from 3 min to 10 h, irrespective of whether night sleep or daytime activities intervened encoding and test. However, retrieval accuracy increased from 3 min to 10 h in both the original and the replication experiment with unconscious encoding if night sleep intervened encoding and test. Hence, the consolidation trajectories of consciously and unconsciously acquired episodic memories went into opposite directions.

The 10 h retention of subliminally presented cartoon clips seems unexpected given the literature on subliminal processing indicating that subliminal stimuli may affect human behavior over 1 s at most ^18–23^. Because these short-lived subliminal retention effects were obtained with single-item priming on familiar stimuli such as words, which recruits the neocortex alone rather than the hippocampus^5^, the resulting neocortical memory traces decay rapidly. When the stimulus material is complex, involving many novel items for associative binding (as given for the cartoon clips), then the encoding process recruits the episodic memory system including the hippocampus with its fast-acting plasticity mechanisms that enable a long-term storage^5–9,12^. The finding of a retention of subliminally encoded clip contents over 10 h, at which time they guide the outcome of deliberate human decisions, is unprecedented. This finding reveals powerful and long-lasting influences of unconsciously acquired information on conscious human behavior. Because the encoding process is effortless and the behavioral modulation occurs without the notice of the subject, this method may bear practical implications for education, behavioral change and clinical research.

Most interestingly, unconsciously acquired episodic memories were not behaviorally relevant immediately following encoding but steered the deliberate decisions made at test only hours following encoding. This was contrary to conscious encoding that yielded memory representations strong enough to affect deliberate decisions immediately. The lack of an immediate behavioral effect of unconscious encoding is owed to the strong masking, which results in a very faint input signal and poor brain activation^12^, and to the hippocampus-dependent processing of the complex associative material^61–63^. In contrast, the weak masking applied for conscious encoding results in a strong activation of the visual cortex and upstream neocortical regions^12^, with good neural propagation and long-range coherence^64,65^. We assume that subliminal episodic encoding that relies on the hippocampus requires an immediate and delayed sleep-dependent functional and structural consolidation to strengthen the memory-underlying neural traces. In fact, a growing body of research focusing on the processing of weakly, but consciously, encoded memories suggests that the weaker the memory representations are following encoding, the more they are in need of sleep consolidation^36–41,66^. For example, Cairney et al.^36^ found that targeted memory reactivation during sleep provided the greatest benefit for the delayed recall of picture-location-in-space memories if these memories were retrieved with a low degree of accuracy prior to sleep. Similarly, when paired-associate word encoding was difficult because words were abstract rather than concrete, the increased difficulty resulted in a poor pre-sleep cued recall but provoked an increase in sleep spindles in language-related left frontal cortex, which in turn improved the delayed post-sleep recall of the abstract word pairs^66^. In general, it appears that initially weak memories—resulting from few learning trials or a shallow processing or suppression instructions or considerable interference—appear to profit more from sleep-dependent memory consolidation than initially strong memories^36–41,66^. Hence, fresh memories that were initially weak because of other reasons than subliminal presentation were prioritized during sleep consolidation. Similarly, unconscious memories formed based on the strongly masked cartoon clips may have had privileged access to consolidation during night sleep, which bolstered their underlying memory traces so the memories could influence decisions at the 10 h retrieval.

Interestingly, the prioritization of weak over strong memories in the consolidation process is not bound to consolidation during sleep but has also been found for wake consolidation: Schapiro et al.^28^ showed with functional magnetic resonance imaging that an initially poor versus good recall of an object provokes more hippocampal replay during ensuing wake rest for that object. In addition, Jafarpour et al.^67^ decoded brain activity from magnetoencephalography data and found that replay during a wake maintenance period favored memories formed with little attention and this replay improved their subsequent retrieval. Finally, Tambini et al.^68^ demonstrated that targeted memory reactivation during post-encoding wakefulness prioritized the weakest memories. Because we had included both wake and sleep consolidation periods in the present study, we could compare the two conditions and found that weak memories profit more from sleep- than wake-consolidation. This is in line with an innovative optogenetic study in mice demonstrating the imperative need of fresh hippocampal memories for synaptic growth during the minutes following encoding and these memories’ need for their encoding-related activity patterns being replayed in the hippocampus during subsequent sleep, while the replay during wakefulness was not effective^14^. Whether initially weak versus strong memories were more dependent on these time-windows of memory consolidation was not investigated.

We would like to point out that memories formed from subliminal cartoon clips are both *unconscious* memories and *weak* memories, the two attributes being confounded by nature. Consequently, the unconsciousness of the memories or their weakness or both attributes may have generated the inverse forgetting curve. Of note, retrieval performance also increased during sleep consolidation for weakly but consciously encoded episodic memories^39,41^. Hence, the weakness alone (without unconsciousness) is sufficient to produce an overnight increase in retrieval performance. We suggest that unconsciously formed episodic memories are much weaker than consciously formed weak memories. This increase in weakness might put unconscious episodic memories at the forefront for a privileged memory consolidation during sleep that yields an inverse forgetting curve. Future research should test this hypothesis.

The longevity of a memory does not only depend on the memory’s access to the brain’s consolidation machinery but also on the degree of associative interference of the memory with previously and subsequently encoded similar memories^69–72^. Interference-based forgetting accounts for a large percentage of inaccessible memories^73^. To our knowledge, interference-based forgetting has only been studied using supraliminal presentations of the learning material, which provides a strong input signal that excites large neural assemblies. Because many hippocampal neurons are activated to some degree during the encoding of several stimuli in a learning list^74^, the resulting neural memory traces overlap, which breeds interference and forgetting^69–72^. This is reflected in the 25% forgetting rate between the 3 min and 10 h recall in the present study. On the other hand, subliminal encoding appears to be almost interference-free as suggested by the inverse forgetting curve obtained in the current study and by the absence of forgetting in spite of a high memory load in a previous study using the same cartoon clips^12^. Because of a weak input signal, the neuronal memory traces coding for individual unconscious memories might perhaps be sparser with distinct neurons coding for distinct memories (not examined yet). This sparse coding would lead to non-overlapping memory traces and hence to less interference and forgetting. Although hypothetical, this scenario resonates with views in computational neuroscience suggesting a larger memory capacity but poorer retrieval fidelity for sparsely (perhaps unconsciously) versus thickly (perhaps consciously) coded memories^75–78^. To conclude, the enhanced unconscious retrieval accuracy at 10 h versus 3 min post-encoding may originate (1) in neural consolidation processes favoring weak memories and (2) in a sparse coding that counteracts interference.

While night sleep versus daytime activities benefitted the consolidation of subliminally formed memories, the two consolidation conditions yielded no differential effects for the retention of consciously encoded clips. This null-effect coincides with many other reported null-effects that together question the key findings of sleep-mediated memory benefits^79^. For example, no significant correlations between episodic memory retrieval and parameters of slow-wave sleep were found in a study including 885 participants^80^. Advantageous effects of sleep on the retention of consciously encoded episodic memories remain if the learning situation featured these properties: (1) participants need to know before going to sleep that their memory will be tested later^81^ and only the memories intended for retrieval improved through sleep^82^; (2) corrective feedback or restudy must be included in the learning situation^83^; (3) post-sleep interference learning must be implemented before retrieval testing^84,85^. Because none of this applied to the current design, we may have missed a beneficial effect of sleep on the retention of consciously encoded clips.

The design of the present encoding task puts high demands on visuospatial working memory. This same task elicited activation in regions of the right prefrontal cortex, which underlies visuospatial working memory^12^. Therefore, we had anticipated that participants would need a good visuospatial working memory to tackle this difficult encoding task. Although this held true for the situation when encoding was unconscious, the quality of visuospatial working memory appeared irrelevant for the conscious encoding of the clips. This is reminiscent of the tendency of subliminal information to be processed through the right cerebral hemisphere^86,87^, whereas supraliminal information is verbally coded and therefore processed through the left hemisphere^88^. Hence, when clip encoding was conscious, participants might have employed a verbal form of coding the animals and their trajectories, which recruited verbal in addition to visuospatial working memory and hence the left prefrontal cortex. Indeed, Schneider et al.^12^ report that conscious versus unconscious temporal relational inference making during clip encoding was paralleled by left more than right signal increases in Broca’s area in the inferior frontal gyrus. This suggests that participants might have employed a verbal form of coding the animals and their trajectories. To conclude, because the drawing of inferences requires working memory^12,48–53^, only participants with a strong working memory were able to encode the essential clip information unconsciously. Participants with a lower working memory capacity were probably unable to draw inferences unconsciously and hence lacked the essential clip information which the retrieval test asked for.

The present findings evidence an unconscious form of working memory and episodic memory. This extends theories that associate working memory with conscious stimulus processing^43,89,90^ and theories of consciousness that consider conscious scene perception necessary for the encoding of an unfolding event including its what-where-when aspects^90–95^. The findings confirm theoretical claims^13,16,96^ that the premise for episodic encoding is not consciousness but a task that calls upon the core computational competence of the episodic memory system, which is the rapid formation of new and flexible associations.

Although new episodic memories are formed with and without conscious awareness of the encoding material, the encoding and consolidation processes appear to differ between levels of consciousness: conscious encoding yields memories with an immediate and strong impact on human behavior, but the capacity of conscious memory is limited, with memories interfering and dissipating. Unconscious encoding on the other hand yields initially weak and little-interfering memories that are in an imperative need of an immediate and sleep-dependent consolidation to strengthen to the point where they affect human behavior. Our findings attest to the powerful and long-lasting influence of unconscious memories on human decision-making.

## Methods and material

The current research consists of three experiments that have implemented the same memory task. This task involves the encoding of short cartoon clips that needed to be stored for later retrieval (Figs. 1 and 3). In the first and second (replication) experiment, the presentation of the movie clips was subliminal for unconscious encoding. Participants were kept naïve to the fact that they are exposed to subliminal information. Participants performed an attention task superimposed upon the subliminally presented clips (Fig. 1B). In the third experiment, the presentation of cartoon clips was supraliminal for conscious encoding. Participants were fully aware of the clips and the learning situation. Retrieval testing for a given clip was prompted either after clip offset or three minutes later or 10 h later. In the latter case, the study-test interval comprised either a night of sleep or a day filled with daytime activities (Fig. 1C). The cartoon clips were presented strongly masked for subliminal (unconscious) encoding and weakly masked for supraliminal (conscious) encoding.

### Experimental tasks

#### Encoding

The clips featured five animals that traversed a scene one-by-one from left to right or from right to left. The background depicted a specific landscape scenery of a habitat including a hiding place (e.g. a shipwreck on the bottom of the ocean). The five animals (e.g. shark, octopus, turtle…) moved across the scenery towards the centrally positioned hiding place and disappeared inside the hiding place. At any one time, only one animal was moving towards the hiding place or was hiding inside the hiding place or was leaving the hiding place to exit the scenery. Once an animal was inside the hiding place, it could not be seen by the viewer anymore, neither in the condition with strong masking nor in the condition with weak masking. An animal could either move through the hiding place and exit it immediately or linger inside the hiding place for an unpredictable dwelling time and meet other animals that had entered before or would arrive later. Hence, while watching a cartoon clip, participants needed to maintain a mental record of which animals lingered simultaneously inside the hiding place based on the temporal sequence of the five animals’ entrances and exits (Fig. 3). This task requires the viewer to draw temporal relational inferences—a cognitive process that requires both working memory and episodic memory.

#### Retention conditions

Three different study-test intervals were implemented (immediate retrieval; retrieval at three minutes following encoding; retrieval at ten hours following encoding). After the presentation of a cartoon clip, retrieval testing was prompted immediately after clip offset or there was a three-minute break. In the latter case, retrieval testing was prompted after break offset or 10 h later. We presented six clips per retention condition, i.e., 18 clips in total. The clips belonging to the three retention conditions were presented in random order rather than block-wise (Fig. 2A). During the three-minute breaks, the screen turned black and participants were asked to relax.

#### Retrieval testing

A test trial consisted of the unmasked, i.e., clearly visible, presentation of a clip’s scenery plus the unmasked images of two animals that had featured in the clip. The participants’ task following supraliminal clip presentations was to decide whether the two presented animals had lingered simultaneously inside the hiding place—yes or no (Figs. 1D and 3). If clip presentations were subliminal, the task was to decide intuitively whether the two animals would linger simultaneously inside the hiding place in a fictitious clip. After every retrieval trial, participants rated on a 4-point scale the confidence in their response (supraliminal condition) or the felt ease of their response (subliminal condition) (Fig. 1E). We gave ten retrieval trials per clip because we presented each of the five acting animals with every other animal. The retrieval part was self-paced in all experiments.

### Stimuli

We used 19 cartoon clips, of which one was solely used for the practice runs given before experimentation, and 18 were used for the experiment. Each clip featured colored, cartoon-style images of a landscape scenery plus five animals that naturally belong to the shown habitat. The landscape scenery could be an underwater scene, a mountain scene, a farm scene, etc. For each of the 18 experimental sceneries, we had two different sets of five animals, such that the scenery could be shown twice with either set of protagonists. In the subliminal version of the experiment with strong masking, each landscape scenery was indeed presented twice with distinct sets of animals: first in the actual experiment and then in the objective awareness test that followed the experiment. The two distinct sets of animals per landscape scenery used in the experiment and the awareness test were counterbalanced across participants to balance the animals shown in a landscape scenery between the experiment and the awareness test. When the masking was weak for conscious clip processing and no objective awareness test followed the experiment, we still presented both sets of animals for each landscape scenery within the experiment to counterbalance the animals per landscape scenery across participants, as it was done in the strongly masked version of the experiment.

Each clip consisted of 40 individual frames. A frame depicted either the landscape scenery and a single animal that moved towards or away from the hiding place or the landscape scenery alone, featuring only the hiding place. An animal moved across the scenery to reach the hiding place in three frames. Another three frames showed the animal leaving the hiding place and exiting the scenery. When an animal had reached the hiding place or had exited the scenery, the hiding place alone was displayed. For the 18 cartoon clips, we had the five animals cross the scene and meet other animals inside the hiding place in 18 distinct versions of animal trajectories. Hence, no clip corresponded to any other clip regarding the sequence of animals, their dwelling time inside the hiding place, and their encounters with other animals. This counteracted implicit sequence learning. The sequence of animals’ entrances, exits and the dwelling times for a specific scenery was not matched between the main experiment and the objective awareness test.

### Clip presentation

We adopted and modified the masking protocol used by Degonda et al.^6^. Clip frames (F) were presented for 17 ms and masks (M) for 183 ms. Each clip frame was flanked by masks and repeated three times in sequence before the next clip frame was flashed to continue an animal’s trajectory. Five adjacent masks initiated a clip and one extra mask terminated a clip. Accordingly, the frame order in a clip was M–M–M–M–M–F1–M–F1–M–F1–M (…) M–F40–M–F40–M–F40–M–M. For subliminal presentation, we used pattern masks consisting of randomly arranged colored pixels to induce a strong masking effect that impedes a conscious frame perception. Because pattern masks interrupt the neural responses to flashed frames, the processing of the frames does not reach consciousness^97^. For supraliminal presentation, masks were uniformly gray to induce very weak masking that still allows for the conscious perception of each clip frame. A complete clip consisted of 120 clip frames plus 126 masks and lasted 25.1 s.

### Technical setup

Clips were presented with a Benq© MX764 DLP video projector that had a resolution of 1920 × 1080 pixels and a screen refresh rate of 60 Hz. Clips were projected onto a rear projection screen (PLEXIGLAS® Optical by Evonik Industries) with a viewing angle of 16° width and 9° height. We programmed the experiments in Presentation Version 20.3^98^. Responses were logged using a response pad.

### Betsch personality inventory

We used the Betsch *Preference for Intuition and Deliberation* inventory^99^ to assess participants’ habitual decision style. Each participant filled out the inventory prior to experimentation. The Betsch inventory consists of two independent subscales that measure the habitual preference to use intuition or deliberation when making decisions. Because the individual habitual decision style had no influence on the present results, we do not report on this measure.

### Measures of working memory capacity

Before experimentation, we collected a measure of the participants’ visuospatial working memory capacity (Fig. 1A), which is critical for the encoding of the presented cartoon clips. The Corsi block-tapping backwards task was our measure of visuospatial working memory^100,101^. Participants needed to repeat a given sequence of pointed-out blocks in reverse order on a board filled with blocks. The block span is defined as the highest number of blocks correctly repeated in two trials.

### Subliminal experiments with strong masking for unconscious encoding

#### Participants

In the first experiment with strong masking, a total of 73 young women and men were tested. Five participants were excluded from the analyses due to compliance failure with given instructions. Those participants displayed a strong bias in their response behavior at retrieval testing, using predominantly one button only (exclusion criteria: + /− 1.5 std). Therefore, 68 participants were included in the statistical analyses (age: 23.79 (mean) ± 3.54 (std), range: 19–37; 78% women, day group: n = 37, night group: n = 31). In the replication experiment, a total of 36 young women and men were tested, who were exclusively high-working-memory performers. Five participants were excluded from the analyses due to a bias in their response behavior at retrieval testing. Hence, 31 participants were included in the statistical analyses (age: 22.51 (mean) ± 2.83 (std), range: 19–29; 71% women, day group: n = 15, night group: n = 16). Participants reported normal or corrected-to-normal visual acuity. Participants were recruited via an online platform of the student body run by the University of Bern. Participants were kept naïve to the subliminal stimulation protocol and were only debriefed before the objective awareness test. All participants gave their written, informed consent prior to experimentation and they were reimbursed at the end of the experiment. The study was approved by the ethics committee of the Faculty of Human Sciences of the University of Bern. All Experiments were performed in accordance with relevant guidelines and regulations.

#### Experimental procedure

Depending on the experimental group (day group or night group), participants were invited to the laboratory in the morning or in the evening. Upon arrival, measures of visuospatial working memory were collected (Fig. 1A). To keep participants naïve to subliminal stimuli and to conceal the true aim of the study, a cover story was conveyed. Participants were told that we sought to examine the influence of visual depletion and sleep on speculative behavior. Next, the participants rated all animals and landscape sceneries that would later appear in the cartoon clips in order to facilitate their subliminal sensory processing in the experiment. For this rating, we presented each of the 90 animals and each of the 18 landscape sceneries one-by-one on a computer monitor in random order. Participants were asked to indicate by button press whether they liked or disliked the picture. To establish a processing routine (a task set) for the upcoming experimental task, two supraliminally (visibly) presented cartoon clips were presented for conscious encoding, each followed by immediate retrieval testing. The two clips contained the same scenery but distinct sets of five animals (clip number 19 that served only as demo clip). A retrieval accuracy of 60% or more was required for the second clip, otherwise the clip presentation and retrieval testing was repeated. Next, the attention task was introduced to participants because participants performed this attention task in the experiment while the subliminal clips were being presented. Participants were told (as part of the cover story) that this attention task would serve the induction of a visual depletion in the experiment. The visual pattern masks that flanked the frames of a cartoon were centrally superimposed with a small but salient yellow cross (Fig. 1B). Four to five times during the presentation of a movie clip, one of the yellow line segments of the cross was removed resulting in either a single yellow vertical or a single yellow horizontal line segment. Participants were instructed to press the left button as quickly and accurately as possible when the cross turned into a single horizontal line segment and the right button when the cross turned into a single vertical line segment. The last part of the practice runs concerned retrieval testing. Participants were told that they would now practice speculative decisions. The picture of a landscape scenery was displayed on the screen and participants were instructed “Imagine a fictitious animal cartoon. Speculate whether the two animals that are going to be presented would linger simultaneously inside the depicted hiding place—yes or no. Rely on your intuition.” Ten animal pairs were presented for participants to give a yes or no response. To counteract biases in response behavior, participants were instructed to evenly balance “yes” and “no” responses over the experiment, while still relying on intuition (Figs. 1D and 3). Following every trial, participants rated on a 4-point scale their felt ease to respond (1: easy; 2: rather easy; 3: rather difficult; 4: very difficult) (Fig. 1E). Then, the actual experiment started. Participants were briefed as follows: “You will now go through the attention task 18 times. Sometimes, there will be a break of three minutes between two attention tasks and sometimes there will be no break between two attention tasks. The decision-making task may or may not appear following an attention task or following a break.” Next, participants belonging to the day group were reminded of the second experimental session that would take place 10 h later in the evening of the same day. Participants belonging to the day group were asked not to take any daytime naps. Participants belonging to the night group were tested in the evening and asked to return to the laboratory 10 h later on the next morning. Participants belonging to the night group were instructed to sleep at least seven hours and not to drink any alcoholic beverages. Participants belonging to the night group were given an actigraphy device^59^ to measure the ambient brightness and movement-related behavior. During the second experimental session, participants went through retrieval testing concerning the six remaining cartoon clips. Then, the objective awareness test followed (Fig. 1F). We fully debriefed the participants before they took the objective awareness test. In particular, we informed them of the subliminal cartoon clips that had been presented during the attention task. Next, we familiarized the participants with the new sets of animals that featured in the landscape sceneries used in the objective awareness test (because this was also done before the experiment). Hence, participants rated all sceneries and animals that were going to be presented subliminally in the objective awareness test. They simply indicated whether they liked or disliked the picture.

#### Objective and subjective measures of clip awareness

An objective test of clip awareness was administered following the subliminal experiments that featured strongly masked cartoon clips. First, participants were fully informed of the true nature of the experiment including the fact that subliminal cartoon clips had been presented during the attention task. The objective awareness test was performed to scrutinize the effectivity of the strong masking procedure. In the objective awareness test, we re-presented the landscape sceneries that we had presented in the experiment. Yet, we presented the landscape scenery with a new set of five animals. The sequence of animals’ entrances, exits and the dwelling times for a specific scenery was not matched between the main experiment and the objective awareness test, i.e. the choreography of trajectories did not correspond. Eighteen cartoon clips were presented in the awareness test. Retrieval testing took place immediately following a cartoon clip (Fig. 2B). The cartoon clips were presented in the same strongly masked fashion and with the same attention task and the same psychophysical set-up as during the experiment. Because participants were now told that subliminal cartoon clips would be presented, we could give direct retrieval instructions (retrieval instructions in the main experiment were indirect). The difference between instructions (direct versus indirect) was the only difference in the procedure between the experiment and the awareness test. This difference is thought to reveal any potential conscious clip processing. The direct instruction asked the participants to consciously and deliberately decide, based on what they might have consciously seen, whether the two animals presented on a retrieval trial were simultaneously inside the hiding place, yes or no. We also collected subjective data regarding clip awareness. To this end, we asked participants to score the visibility of each subliminal cartoon clip following its presentation on a 4-point perceptual awareness scale^60^ with the levels: (1) no awareness at all; (2) a feeling that something was present, either static or moving, (3) an impression of the scene or animals, (4) a clear image of the scene and animals.

### Supraliminal experiments with weak masking for conscious encoding

#### Participants

We tested 36 young women and men (age: 23.31 (mean) ± 4.17 (std), range: 19–38; 86.1% women) who reported normal or corrected-to-normal visual acuity. Participants were recruited via an online platform of the student body run by the University of Bern. The day group included 19 and the night group 17 participants. All participants gave their written, informed consent prior to experimentation. Participants were fully debriefed and reimbursed at the end of the experiment. The study was approved by the ethics committee of the Faculty of Human Sciences of the University of Bern. All Experiments were performed in accordance with relevant guidelines and regulations.

#### Experimental procedure

The procedure of the supraliminal experiment with weak masking differed in the following ways with respect to the subliminal experiment with strong masking. Because clip presentation was supraliminal, there was no need for a cover story. The attention task during clip presentation was removed. Thus, the practice runs consisted merely of two supraliminally presented animal cartoons. Participants needed to reach an accuracy score of at least 60% in the second run in order to proceed to the experiment. Clip images were now weakly masked using grey masks, which resulted in a conscious perception of the clips (Fig. 1B). Therefore, participants were fully aware of the learning situation. However, to ensure an incidental retrieval situation during the second experimental session and to eliminate any rehearsal during the 10 h study-test interval, participants were told that they would be presented with new cartoon clips on the second experimental session. The retrieval instruction was as follows: “Did the two here presented animals linger simultaneously inside the depicted hiding place, yes or no?” After every retrieval trial, participants rated on a 4-point scale the confidence of their response (1: very confident; 2: rather unconfident; 3: rather unconfident; 4: unconfident) (Fig. 1E).

## Supplementary Information

Supplementary Information 1.

Supplementary Information 2.

## Data Availability

The data generated and analyzed during this study are included in the supplementary information files.
