# Supplementary material for: Inverse forgetting in unconscious episodic memory
Source: Sci Rep. 2022 Nov 29;12:20595. doi: 10.1038/s41598-022-25100-w (PMC9709067; doi:10.1038/s41598-022-25100-w)
Supplement: Supplementary file 2 — Supplementary Information 2. [file 41598_2022_25100_MOESM2_ESM.docx]

**Inverse forgetting in unconscious episodic memory**

Luca Pacozzi, Leona Knüsel^1^, Simon Ruch & Katharina Henke

### **Supplemental information**

### **Objective test of clip awareness and subjective awareness ratings**

When the main experiment featured strongly masked cartoon clips for unconscious encoding, we had participants take an objective test of clip awareness following the 10-hour retrieval to assess the effectiveness of the applied masking paradigm. Following the main experiment, we informed the participants that subliminal cartoon clips had been presented in the main experiment and would again be presented in the objective test of clip awareness. With participants informed of subliminal clips, the encoding and retrieval instructions in the objective awareness test were direct (rather than indirect as in the main experiment). In the objective awareness test, we presented a new set of 18 cartoon clips using the strong masking protocol applied in the main experiment. As in the main experiment, participants performed a central attention task during the presentation of the masked clips. Immediately following the presentation of a clip, participants were asked to rate their subjective visual awareness of the clip on a 4-point perceptual awareness scale (PAS) [1] with the levels: 1) no clip awareness at all; 2) a feeling that something was present, either static or moving, 3) an impression of the scene or animals, 4) a clear image of the scene and animals. Following this subjective awareness rating, we presented the 10 retrieval trials concerning the just presented cartoon clip. As in the main experiment, we displayed in each retrieval trial the hiding place plus two animals (with no masks) and required participants to decide - based on what they might have seen - whether the two presented animals lingered simultaneously inside the hiding place or not. Hence, the objective test of clip awareness differed from the main experiment in two ways: First, retrieval trials followed the clip presentation immediately and second, participants were no longer naïve regarding subliminal clips and were instructed explicitly/directly. Explicit retrieval instructions reveal whether clip elements are potentially consciously perceived.

#### **Original experiment with strong masking**

*Objective measure of clip awareness*: We report the results of the high-working-memory performers because only those participants exhibited a mean retrieval accuracy that was better than chance. The high-working-memory performers’ mean accuracy on the objective test of clip awareness was 51.03% (95% CI [49.76, 52.31]), which was not significantly above chance level (*t* (26) = 1.663, *p* = .108, two-tailed). The Bayes Factor was BF_01_ = 0.48, which indicates anecdotal evidence for H_0_. Neither the day nor the night retention subgroup displayed a mean retrieval performance above chance level (Bonferroni-adjusted, α = .025, day group: 50.87%, 95% CI [49.30, 52.43], t (15) = 1.183, *p* = .255, two-tailed, BF_01_ = 0.29; *night group*: 51.27%, 95% CI [48.76, 53.78], *t* (10) = 1.126, *p* = .287, two-tailed, BF_01_ = 0.4).

Next, we related the high-working-memory performers’ 10-hour retrieval accuracy to their accuracy scores on the objective test of clip awareness to find out whether their conscious clip perception would predict their implicit retrieval performance in the main experiment. Moreover, the intercept in this regression provides a critical measure of subliminal processing: If the regression intercept crosses zero for the awareness test but is significantly above zero for the experimental task, this is strong evidence for unconscious processing [2]. For this regression, chance-level performance (accuracy of 50%) was subtracted from the accuracy scores on the awareness test and from the 10-hour retrieval scores obtained in the main experiment. Performance accuracy on the objective test of clip awareness did not predict retrieval accuracy in the main experiment (*β* = -.021, *t* (25) = -.154, *p* = .879). The intercept of the regression was above chance with accuracy on the awareness test at 0 (*t* (24) = 2.923, *p* = .007, two-tailed, BF_10_ = 13.8) suggesting unconscious clip processing in the main experiment. We ran this analysis also for the day and night retention group individually. In neither group did the performance accuracy on the objective test of clip awareness predict the 10-hour retrieval accuracy in the main experiment (both *p > .714*). The regression intercept was significantly above 0 in the night group alone (Bonferroni-adjusted, α = .025, *t* (8) = 3.191, *p* = .011, two-tailed, BF_10_ = 30.6).

*Subjective measure of clip awareness:* The subjective visual awareness ratings ranged from 1) no clip awareness, over 2) a feeling that something was present, to 3) an impression of the scene or animals, to 4) a clear image of the scene and animals. A rating of 1was given to 90.52% of all cartoons (Supplementary Table 2). The majority of participants rated all clips with a 1 (54.4%; 51.85% for low-working-memory performers and 56.09% for high-working-memory performers). Importantly, the accuracy scores on the objective test of clip awareness were still at chance level if cartoons with a PAS rating of 1 were excluded from this analysis. This was true for both the entire sample and for the high-working-memory performers. Hence, a subjective feeling of ‘something being present’ does not improve accuracy but may be a consequence of knowing that subliminal clips are being presented.

*Equal levels of attention towards the masked clips in the main experiment and in the objective test of clip awareness*: When clips were presented strongly masked for unconscious encoding (in the main experiment and in the objective test of clip awareness), participants performed a central attention task to ensure their continued central visual fixation and visual attention. Accuracy on the attention task performed during the objective test of clip awareness did not differ from the accuracy obtained in each of the 3 retention conditions of the main experiment (paired-samples t-tests: Bonferroni-adjusted α = .016, all *p >* .483, two-tailed). Hence, the level of attention devoted to the processing of the strongly masked clips did not differ between the main experiment and the objective test of clip awareness.

#### **Replication experiment with strong masking**

*Objective measure of clip awareness*: The high-working-memory performers’ mean accuracy on the objective test of clip awareness was 50.15% (95% CI [48.91, 51.38]) and did not differ significantly from chance (*t* (30) = .242, *p* = .810, two-tailed, BF_01_ = 0.123). Neither the day retention subgroup (50.82%, 95% CI [49.22, 52.42]) nor the night retention subgroup (49.51%, 95% CI [47.53, 51.50]) displayed accuracy scores above chance level (*day group*: *t* (14) = 1.099, *p* = .290, two-tailed, BF_01_ = 0.266; *night group*: *t* (15) = -0.522, *p* = .609, two-tailed, BF_01_ = 0.209). Hence, we can assume that participants were not able to consciously perceive the cartoon clips.

Next, we related the participants’ (all high-working-memory performers) 10-hour retrieval accuracy to their accuracy scores on the objective test of clip awareness to find out whether their conscious clip perception would predict their implicit retrieval performance in the main experiment. Performance accuracy on the objective test of clip awareness did not predict retrieval accuracy in the main experiment (*β* = .317, *t* (29) = .879, *p* = .386). The intercept of the regression was marginally above chance, when accuracy on the awareness test was at 0 (*t* (28) = 1.823, *p* = .079, two-tailed, BF_01_ = 1.14). We ran this analysis also for the day and night retention group individually. In neither group did the performance accuracy on the objective test of clip awareness predict the 10-hour retrieval accuracy in the main experiment (both *p > .287*). The regression intercept was significantly above chance in the night group alone (Bonferroni-adjusted, α = .025, *t* (13) = 2.598, *p* = .021, two-tailed, BF_10_ = 5.2).

*Subjective measure of clip awareness:* 41.9% of participants rated all clips with a 1 (*night group*: 62.5%; *day group*: 20%). Importantly, the accuracy scores on the objective test of clip awareness were still at chance level if cartoons with a PAS rating of 1 were excluded from this analysis. Hence, a subjective feeling of ‘something being present’ does not improve accuracy but may be a consequence of knowing that subliminal clips are being presented.

*Equal levels of attention towards the masked clips in the main experiment and in the objective test of clip awareness*: Accuracy on the attention task performed during the objective test of clip awareness did not differ from the accuracy obtained in each of the 3 retention conditions of the main experiment (paired-samples t-tests: Bonferroni-adjusted, α = .016, all *p >* .360, two-tailed). Hence, the level of attention devoted to the processing of the strongly masked clips did not differ between the main experiment and the objective test of clip awareness.

**Supplementary table 1: Overview of behavioral results**

*Notes:* This overview table shows descriptive statistics for the entire samples and for the subgroups in the three reported experiments. OAT, objective awareness test; Day, retention during daytime activities; Night, retention during night sleep; N, number of participants; Accuracy, percentage of correct responses; Δ RT, reaction time of incorrect trials minus reaction time of correct trials; M, mean; SEM, standard error of mean.

**Supplementary Table 2: Results of the perceptual awareness scale (PAS)**

*Notes:* PAS, Perceptual Awareness Scale; M_Ratings(%)_*,* mean percentage of clips rated at this level; SD, standard deviation of the mean.

**Supplementary Table 3: Distribution of working memory capacity scores**

| **Weak Masking** |  |  |  |  |  |  |  |  |
| --- | --- | --- | --- | --- | --- | --- | --- | --- |
|  | **capacity** | **2** | **3** | **4** | **5** | **6** | **7** | **total** |
|  |  |  |  |  |  |  |  |  |
|  |  |  |  |  |  |  |  |  |
|  | n | 0 | 2 | 9 | 9 | 11 | 5 | 36 |
| Non-verbal wm | mode |  |  |  |  |  |  | 6 |
|  | median |  |  |  |  |  |  | 5 |

| **First experiment with strong Masking** |  |  |  |  |  |  |  |  |
| --- | --- | --- | --- | --- | --- | --- | --- | --- |
|  |  |  |  |  |  |  |  |  |
|  | n | 0 | 8 | 13 | 20 | 14 | 13 | 68 |
| Non-verbal wm | mode |  |  |  |  |  |  | 5 |
|  | median |  |  |  |  |  |  | 5 |

Note: Distribution of the working memory scores (Corsi block tapping backwards) for the experiment with weak masking and for the first experiment with strong masking.

References

1. Overgaard, M., Rote, J., Mouridsen, K., & Ramsøy, T. Z. (2006). Is conscious perception gradual or dichotomous? A comparison of report methodologies during a visual task. *Consciousness and cognition*, *15*(4), 700-708.
2. Greenwald, A. G., Draine, S. C., & Abrams, R. L. (1996). Three cognitive markers of unconscious semantic activation. *Science*, *273*(5282), 1699-1702.
